# Web-Based Training for Primary Healthcare Workers in Rural China: A Qualitative Exploration of Stakeholders’ Perceptions

**DOI:** 10.1371/journal.pone.0125975

**Published:** 2015-05-11

**Authors:** Zhixia Zhang, Xingxin Zhan, Yingxue Li, Rong Hu, Weirong Yan

**Affiliations:** Department of Epidemiology and Biostatistics, School of Public Health, Tongji Medical College, Huazhong University of Science & Technology, Wuhan, Hubei, P.R. China; TNO, NETHERLANDS

## Abstract

**Background:**

Equitable access to basic public health services is a priority in China. However, primary healthcare workers’ competence to deliver public health services is relatively poor because they lack professional training. Since the availability of web-based training has increased in China, the current study explored stakeholders’ perceptions of a web-based training program on basic public health services to understand their thoughts, experiences, and attitudes about it.

**Methods:**

Six focus group discussions with primary healthcare workers and three with directors of township hospitals, county-level Health Bureaus, and county-level Centers for Disease Control and Prevention were conducted in Yichang City during 2013. Semi-structured topic guides were used to facilitate qualitative data collection. Audio recordings of the sessions were transcribed verbatim and theme analysis was performed.

**Results:**

Most of the study’s participants, especially the village doctors, had insufficient knowledge of basic public health services. The existing training program for primary healthcare workers consisted of ineffective traditional face-to-face sessions and often posed accessibility problems for the trainees. Most of the study’s participants had a positive attitude about web-based learning and expressed a strong desire to receive this novel training approach because of its flexibility and convenience. The perceived barriers to utilizing the web-based training method included poor computer literacy, lack of personal interaction, inadequate infrastructure, and lack of time and motivation. The facilitators of this approach included the training content applicability, the user-friendly and interactive learning format, and policy support.

**Conclusions:**

Web-based training on basic public health services is a promising option in rural China. The findings of the study will contribute knowledge to implementation of web-based training in similar settings.

## Introduction

China is currently reforming its healthcare system. In April 2009, the Chinese central government launched a new round of health reform. One of its five priorities was to offer all residents equitable access to basic public health services (BPHS) [1]. Public health services are the health products and services provided by health administrative authorities and medical health institutions at all levels for health protection and promotion [2]. BPHS are delivered through a three-tiered system of village clinics, township hospitals, and county hospitals. The major objectives of BPHS were to improve the ability of the three-tiered system to strengthen disease control and health promotion and to provide a package of public health services for all residents free of charge[3]. The package consisted of eleven categories of national BPHS, including health records management for residents; health education; healthcare for children under the age of six; maternal healthcare; healthcare for the elderly; immunization; reporting of infectious diseases and public health emergencies; healthcare management of patients with hypertension, type 2 diabetes, or severe mental illness; and health supervision assistance [4].

Primary healthcare workers (PHCWs) include village doctors from village clinics, township health workers from township hospitals in rural areas, and community healthcare workers from community health centers in urban areas. These workers play an increasingly important role in delivering BPHS in China [5]. They provide immunization, maternal health care, health management, and other public health services to rural residents. In addition, village doctors deliver medical services to rural residents [3, 5]. The number of qualified healthcare providers in the primary healthcare workforce affects healthcare delivery, especially service quality in rural areas [6, 7]. Previous studies revealed that the competency of PHCWs in China was relatively poor [6–8] and some BPHS delivery was at a lower level of quality than expected [8].

Training, developed by local health departments (usually county-level Centers for Disease Control and Prevention or county-level Health Bureaus) for PHCWs, was commonly held in regular training seminar format and concentrated in conference sessions[9, 10]. The traditional face-to-face method was the main training method used in rural China[9]. Before 2009, most of the training focused on disease treatment, clinical skill, and health policy introduction[9]. Relatively little attention was paid to training public health services, especially in rural areas [6]. To achieve the goal of equitable access to BPHS, the government has invested funding in primary health care training [6]. Training topics were mainly theoretical knowledge and delivery methods of BPHS [11]. This training remained inadequate and urgently needed [8, 12].

Since the advent of the world wide web in 1991, internet use has increased; its potential as an instructional tool was quickly recognized [13]. Developing countries healthcare workers’ increased access to the internet at all levels and the rapid growth of computer processing has provided excellent opportunities to develop healthcare worker training, to upgrade healthcare services, and to strengthen healthcare systems [14]. Web-based training has the potential to enable global access to the very best educators. It is also more cost-effective than face-to-face educational programs [14]. It permits health professionals to learn at flexible times, remaining in their clinics without traveling [15, 16]. By using the web, different learning styles can be addressed and distance learning becomes possible [17]. Moreover, web-based training has the potential to tailor instruction to individual learners’ needs and to enable all learners to acquire the competencies they need [18].

At the present time, a growing number of educators are experimenting with this innovative learning approach to facilitate the dissemination of heath information and medical knowledge in a variety of low- and middle-income countries [19–22]. The findings suggest that web-based learning has made an effective contribution to the improvement of learning outcomes and could be used for training healthcare professionals in developing countries [14, 19, 20, 23, 24]. At the same time, an increasing number of educators are sharing their experiences in internet use for physician training, healthcare education, and medical student education in China [23, 25–31]. However, few studies have explored the application of the internet to public health training in rural China.

Focus group discussion (FGD), a method used in qualitative investigations, is useful for generating rich, comprehensive, and detailed data that cannot be obtained through quantitative surveys alone. For successful training uptake and implementation, it is important to explore key stakeholders’ perceptions of the design and delivery of the training method. The selected stakeholders included PHCWs and directors from the local county-level Centers for Disease Control and Prevention (CDC), county-level Health Bureaus, and the township hospitals, who work in the field of public health services’ administration and have direct contact with PHCWs [8, 32, 33]. Therefore, the purpose of this study was to capture stakeholders’ thoughts, attitudes, and training experiences in BPHS, and to indentify barriers and solutions to improve methods of providing information to primary healthcare professionals.

## Methods

### Setting and participants

This qualitative study was conducted in three rural counties of Yichang City, Hubei Province in central China in January and February 2013. The gross domestic product (GDP) of Hubei ranks ninth among 31 provinces, municipalities, and autonomous regions in mainland China. Yichang, a city with approximately 4.08 million residents, ranks second among 13 cities in Hubei Province. Yichang City began the implementation of BPHS in 2009.

Three different rural counties with different levels of economic development (low, medium, and high) in Yichang City were selected for this study. In each county, two towns were selected based on their distance from the county center and their economic development. In each county, three FGDs were held; two groups consisted of PHCWs from the township hospitals and village clinics in the two selected towns (one single FGD in each town) respectively, and one group consisted of the directors from local public health institutions (as mentioned above). Interviewees were purposefully selected on the basis that they were currently providing BPHS in primary healthcare settings (for PHCWs only) or in the BPHS administration field (for directors only). To maximize variation in the sample, interviewees were recruited with a range of demographic characteristics (age, gender, and educational background) to achieve varying perspectives about the topic. All participants were identified and approached by the researchers with help from a head director (from Yichang CDC). They were invited to participate in the study by e-mail or telephone. Nine FGDs, each with 8–9 participants, were conducted. Theme saturation was reached by the end of the ninth FGD.

### Interviews and data collection procedures

Before each scheduled interview, all of the participants were contacted by text messages and reminder calls to attend the interviews at a scheduled time. The first author, a healthcare researcher with a medical education background, conducted all of the interviews. She received training in qualitative methods as part of her doctoral (PhD) studies and has extensive experience in conducting interviews. The second author concurrently kept detailed handwritten notes of nonverbal and verbal communication (quoted material) of the group interactions among the participants [34]. The first author conducted all of the interviews to minimize differences in the influences of interviewers’ professional backgrounds and personal characteristics on the participants’ responses, and thus, to ensure consistency across all of the FGDs [35]. All of the discussions were audio-recorded.

The first author developed the semi-structured topic guide used to facilitate all of the FGDs. The topic guide for the six FGDs consisting of PHCWs was the same. It focused on eliciting their understanding of BPHS, previous training experiences on BPHS, and attitudes about web-based training. The topic guide used in the three FGDs with the directors was essentially the same with a few minor differences. This guide included questions about the directors’ perceptions of the PHCWs’ capacity to provide BPHS, perceptions of the BPHS existing training, and attitudes and concerns about web-based training.

After a brief self-introduction by each participant and an explanation of the purpose of the FGD, the researcher encouraged the participants to speak freely and to reflect, share, compare, and react to the group’s interactions. The use of the topic guide was adjusted gradually according to the participants’ perspectives, in order to increase their openness and obtain more information about their topic. At the end of each FGD, the interviewer provided a brief summary of the discussion to assess the accuracy of her understanding and impressions of the participants’ interactions. The participants were encouraged to clarify any discrepancies [36].

The FGDs with the PHCWs (n = 6) were conducted in a meeting room at each local township hospital. The FGDs with the directors (n = 3) were conducted at each local county CDC. Each FGD lasted approximately 80 minutes. All of the participants were compensated for transportation costs and received refreshments at the FGD; there were no other incentives to participate in the FGDs.

### Data analysis

A pragmatic approach was employed in this study to ensure a suitable fit between the research methods and research questions [37]. Thematic analysis techniques were employed, as described below, in which the transcripts were examined closely to identify themes [37, 38].

All of the recordings were transcribed verbatim and hand-written notes were used to supplement the records. The transcripts were checked for accuracy and completeness and then were analyzed by the first and second authors independently. The recurring viewpoints relevant to the questions were listed and the data were coded line by line to produce initial codes. They were compared for similarities and differences to reform the themes and were collated into relevant themes [38]. Finally, the emerging themes were identified and discussed to ensure rigor and reliability until a consensus on the overall themes was reached. Any data that did not fit the themes were discussed and new themes/subthemes were added when necessary. The constant comparative approach [39] in which researchers move back and forth between the data and emerging themes was used until all data were analyzed. When necessary, the researcher reviewed the raw data and used the self-reflexivity method to ensure that the themes were consistent and coherent. The analysis was performed using the NVivo 8 (QSR International) qualitative data analysis software program. We reported the study consistent with the consolidated criteria for reporting qualitative research (COREQ) checklist (S1 Checklist).

### Ethical considerations

Ethical approval for this study was obtained from the Institutional Review Board of Tongji Medical College, Huazhong University of Science and Technology. Written consent was obtained before the study and each participant was assured that individual comments would remain confidential.

## Results

Nine focus groups consisting of 74 participants were conducted. Among the participants, a third (n = 24) were directors from three counties, 25 were PHCWs from township hospitals, and 25 were PHCWs from village clinics. Among the 24 directors, 11 were from the county CDC, seven from the county Health Bureau, and six from township hospitals. The participant characteristics are presented in Table 1. Of the directors, 62.5% (n = 15) were male, half of them (n = 12) were between 41 and 50 years of age, and 70.8% (n = 17) had an undergraduate or higher educational background. Of the PHCWs from the township hospitals, the majority of them (n = 20) were between 31 and 40 years of age, and more than half of them had a junior college educational background. Almost half (n = 12) of the PHCWs from the village clinics were older than 51 years of age, and the majority (n = 20) of them had a secondary education background level or below.

**Table 1:** Participant demographic characteristics.

Characteristic	Directors n (%)	PHCWs from township hospitals n (%)	PHCWs from village clinics n (%)
**Gender**			
Male	15 (62.5)	16 (64.0)	20 (80.0)
Female	9 (37.5)	9 (36.0)	5 (20.0)
**Age (years)**			
31–40	11 (45.8)	20 (80.0)	5 (20.0)
41–50	12 (50.0)	4 (16.0)	8 (32.0)
≥ 51	1 (4.2)	1 (4.0)	12 (48.0)
**Educational level**			
≤ Secondary*	2 (8.3)	5 (20.0)	20 (80.0)
Junior college	5 (20.8)	14 (56.0)	5 (20.0)
Undergraduate or above	17 (70.8)	6 (24.0)	0 (0.0)

* ≤ Secondary: illiterate or primary school, middle school, high school, or technical secondary school

Five over-arching themes were identified. Three themes reflected the overarching topics consistent with their inclusion in the topic guide: knowledge about BPHS, previous BPHS training experiences, and experiences in web-based training. The other two themes, barriers to and facilitators of web-based training, emerged from the analysis. The fourth theme, “barriers,” contained four subthemes: low computer literacy, lack of interaction in web-based learning, inadequate infrastructure, and lack of time and motivation. The last over-arching theme, “facilitators,” encompassed three subthemes: training content applicability, user-friendly and interactive learning format, and policy support (policies and guidance developed by the Chinese government to reinforce web-based training implementation). The direct quotations of the focus group participants that best illustrate and provide a more in-depth presentation of the themes are provided in the sections below. Only the participants’ occupational roles and genders are included with each quote to maintain their anonymity.

### Knowledge about BPHS

Most of the PHCWs, especially the village doctors, acknowledged that their knowledge of BPHS was limited and disorganized, affecting their routine work. A small number of PHCWs from the township hospitals stated that they were well qualified for their duties and responsibilities because they had many years of work experience. However, when some professionals’ knowledge was probed by asking questions such as “What was the standard management rate of patients with hypertension,” they responded with inaccurate answers.

“The PHCWs in our village clinics are old and less educated. We do not know much about basic public health services. Some of the staff does not even know how to provide health education to the patients with hypertension or type 2 diabetes.”

**(Village doctor, male, FGD 8)**

“We still lack knowledge about public health services. For example, it is difficult for us to do follow-ups for patients with hypertension, type 2 diabetes, and mental disorders. We also know little about children’s health management. We do not know how to provide guidance on infant supplementary food feeding and children's psychological development.”

**(PHCW from a township hospital, female, FGD3)**

The directors in the other three groups also commented that the PHCWs’ knowledge was insufficient and the quality of public health services they provided was suboptimal. Additionally, the directors stated that the village doctors had less knowledge than the township hospital providers did.

“We still lack professional health care providers. Many of the PHCWs have a low-level educational background. As far as I know, some PHCWs were unable to do some essential physical assessments. For example, some of them don’t know the correct method of measuring blood pressure or head and chest circumference of the patients.”

**(Director, male, FGD9)**

“About 70% or more of the village doctors in our county are over the age of 60. To be honest, I think their knowledge is poorer compared to the township hospital providers. Most of them did not know how to provide health education to pregnant women.”

**(Director, female, FGD6)**

### Previous experiences in BPHS training

The participants reported that most of the training sessions on BPHS provided by the local township hospitals or the local county CDC were held using the face-to-face format in conjunction with the regularly scheduled job-related meetings at a fixed location. Only a limited number of personnel had the opportunity to participate in the training because those with busy schedules were unable to leave their work units. The village doctors especially encountered difficulties participating in the county-level training when transportation was needed. At the same time, the directors noted that they also faced difficulties in organizing the training because of time and place constraints.

“Generally speaking, we are busy with our work, so we cannot leave our post. We have little opportunity to attend the training and we are frustrated.”

**(Village doctor, female, FGD5)**

“For some village clinics, there are only two village doctors. If both of them go (to receive the training), there will be no one left to work. It is impractical for both of them to join in the training.”

**(PHCW from a township hospital, female, FGD5)**

“It is difficult for everyone (township hospital personnel) to receive county-level training. The person who participated in the training needs to communicate the training knowledge to the others who can’t join the training.”

**(Director, male, FGD3)**

“It is difficult for us to arrange a suitable training time for the PHCWs. The PHCWs are very busy with their job and it is not easy for them to attend the training at a fixed time and place.”

**(Director, male, FGD6)**

Moreover, the amount of training may not meet trainees’ needs. Although the township hospitals held many scheduled job-related meetings with the PHCWs, most of the meeting time was devoted to coordinating routine work or introducing health policies. Only a limited amount of time remained to deliver BPHS training. The PHCWs complained that the knowledge they received was insufficient due to this.

“We have a job-related meeting every month in our township hospital and the meeting usually lasts half a day (three hours). About two hours are used to arrange the daily work and then the remaining hour (of the time) is used to deliver knowledge. I strongly believe the training time is so limited that I learn little every time.”

**(PHCW from a township hospital, male, FGD3)**

“Given the limited training time, many of the training content areas were taught quickly and did not provide details. It was difficult for me to understand this knowledge in such a short time. At times, I did not even understand half of the (training) content.

**(Village doctor, female, FGD4)**

“It is difficult to deliver comprehensive knowledge of BPHS in the limited training time. I do not remember receiving any training on maternal healthcare and healthcare management of patients with severe mental illness.”

**(Village doctor, female, FGD7)**

Some PHCWs also reported that the face-to-face training did not pay attention to learners’ individual differences.

“The knowledge level of BPHS is diverse among different individuals. Some individuals with a higher knowledge level understood the (training) content better. Some people have poor knowledge levels, especially the older individuals, and it could be difficult for them to understand the content in the limited training time.”

**(PHCW from a township hospital, female, FGD2)**

However, some participants felt that face—to-face training would be helpful to acquire a better understanding of the training since the trainees could interact with each other and the instructors immediately.

### Previous experiences with web-based training

Discussions revealed that only a few participants had web-based training experience. The participants with previous experience of web-based training were the PHCWs from the township hospitals. They stated that the web-based training delivered by a learning platform was mainly about clinical medicine, such as internal medicine and surgery. At the same time, they recognized the convenient, timesaving aspects of online learning and expressed a preference for online training rather than the face-to-face method.

“Last year, we received a web-based training program on clinical medicine. It mainly provided (us) with basic medical knowledge. We could login to the platform by using a designated username and password. The main learning format was to see the video in the platform…In my opinion, we have not received such (web-based) training on BPHS.”

**(PHCW from a township hospital, female, FGD5)**

Although the majority of PHCWs did not have web-based training experience, their discussions suggested their strong willingness to accept this novel training approach. They reported their belief that web-based learning could be an informative and educational tool. They welcomed the advantages of online learning, such as the equal opportunities it offered individual learners, and its ability to overcome geographical limits. They said they would be able to continue working while studying. Some directors commented that web-based learning is productive and cost-effective and avoids facility constraints. Moreover, many of them affirmed they have easy access to a computer and that every primary healthcare institution (both township hospitals and village clinics) is equipped with at least one computer.

“The web-based training sounds new and interesting to me. I think this learning method is convenient since we can learn (by ourselves) in our clinic without traveling. There are 21 village clinics in our town and it is not practical for us to centralize in a fixed location to receive the training often. Besides, web-based learning can also broaden our view.”

**(Village doctor, male, FGD 7)**

“I think web-based learning is a good choice for me. I can study not only in my workplace, but in my home…I will continue my work while studying.”

**(PHCW from a township hospital, male, FGD5)**

“I strongly think web-based training is very important and it is so necessary to establish a learning website for us. In my opinion, everyone can accept this learning method. You can know how much you have learned and self-assess the learning effect.”

**(PHCW from a township hospital, male, FGD6)**

“I think it is feasible to develop a web-based training program in the village, as almost all of the villages are equipped with computers, and it is a good opportunity for all of the village doctors to receive more systematic knowledge so that their ability to deliver public health services will improve accordingly. It is cost-effective as it will take place at their (PHCWs’) health institutions (no accommodations, food, and transport costs).”

**(Director, male, FGD9)**

### Barriers to implementing web-based training

Participants identified several potential barriers to web-based training discussed in this section.

#### Low computer literacy

Most of the PHCWs commented that they grasped basic computer skills, such as surfing on the internet, searching for information, and downloading beneficial learning materials from websites when they discussed computer literacy. However, we found from the discussions that the older PHCWs (those over age 60) had a low level of computer literacy. Some directors explained that more than half of the village doctors were older. The younger generation was unwilling to work in primary healthcare institutions because of the low salaries and fewer opportunities for personal/professional development. Although information management is an indispensable component of providing public health services, some of the older village doctors still lacked skills and knowledge related to computer and internet use. Hence, some of the village doctors expressed a desire for training in computer use.

“Our village clinic is equipped with a computer. There are four healthcare workers in our village clinic, three of them are nearly 60 years old, and they are not able to operate a computer. When they turn off the computer, they just pull the plug…”

**(Village doctor, male, FGD2)**

“As I know, some older village doctors type so slowly that it was just like watching someone catching a worm when they typed on the keyboard. They could not open the web pages, not to mention making a document and sending an email.”

**(PHCW from a township hospital, female, FGD8)**

#### Lack of interaction

Lack of face-to-face contact also was reported as a concern. Some participants who had web-based training experience stated that they felt isolated by online learning. Many PHCWs who did not have online learning experience also revealed that distance online learning would limit their communication with others.

“I think the biggest weakness of web-based learning is lack of interaction. When I encounter something that is difficult for me, I feel frustrated when I do not know whom I should ask (for help).”

**(PHCW from a township hospital, female, FGD8)**

#### Lack of time and motivation

Although web-based training was perceived to be more convenient and flexible compared to the face-to-face format, some of the PHCWs stated that their heavy workload limited the time they could spend in web-based training. Some village doctors commented that they should provide medical services to rural residents and work as farmers to supplement their earnings. Moreover, some village doctors felt that their efforts to provide BPHS were not valued by the government and lacked motivation to receive more training in this field.

“We are very busy. We provide both the public health services and medical services to the residents. Usually, we work at night to complete all the activities. It takes time to study online. It is unlikely for us to see video online.”

**(Village doctor, male, FGD2)**

#### Inadequate infrastructure

Finally, a small number of participants mentioned poor internet connectivity and outdated computers in some village clinics as a concern. They complained that the slow internet speed limited their ability to see videos and download material from the internet easily.

“I do not prefer web-based learning. The internet speed in our village is very slow; it often takes me a long time to download a file from the internet. Sometimes, we cannot even open the videos. Besides, the computer is so old that it runs slowly.”

**(Village doctor, male, FGD4)**

### Facilitators to implementing web-based training on BPHS

#### Training content applicability

Participants across all of the groups expressed the opinion that it was crucial that the content of learning materials be attractive, easy to understand, use professional terminology and avoid jargon, and clarify terms as needed. The most important aspect of the learning situation is that it should be relevant to their routine work. Some of the participants stressed that the content should be comprehensive and tailored to the intended users’ needs at the township and village levels. They said that it should include basic theoretical medical knowledge and professional knowledge and that case studies with real-world examples would be preferable.

“There is a big gap in the knowledge level between the village doctors and the township hospital providers, so the learning materials should be comprehensive. Besides, the content of the learning materials should be easy to understand because it may be easy for the young and difficult for the older generations.”

**(Director, male, FGD1)**

“The training materials should correspond with our daily work. It should be practical and beneficial for us to follow. The course developers should consult real-world experts and practitioners to help develop the learning materials.”

**(PHCW from a township hospital, female, FGD4)**

#### User-friendly and interactive learning format

Participants across all of the groups stressed the need for a user-friendly web-based training platform to make online learning easier for them. It should be designed to be clear, simple, interesting, and easy to use. Many PHCWs said that clarity, navigation, and graphics might attract users’ attention and increase their engagement. Some directors commented that audio clips were especially helpful for people with visual difficulties and that downloadable material and manuals could be offered. Most of the PHCWs and directors stressed that interaction also was a very important factor that should be considered. In order to establish an interactive training platform, certain methods including forums and interactive quizzes that give automatic feedback would assist.

“I think the interaction is very important. If I encounter something difficult, I can communicate with experts instantly. It can also deepen my understanding of content.”

**(PHCW from a township hospital, female, FGD6)**

“I think it would be help if there was a discussion forum or an online chat room in the training platform. By using them (discussion forum or an online chat room), trainees could communicate with fellow students and experts. If the trainees could send personal messages to each other or experts and get immediate feedback, I think their interest in learning would significantly increase.”

**(Director, female, FGD9)**

#### Policy support

Technical and administrative support also was perceived to be a facilitator. The government should attach importance to the training and provide financial support to these primary health institutions. Examples of such support include making a larger investment in improvements of the infrastructure, providing up-to-date computers, and speeding up the broadband.

## Discussion

Data from this qualitative study revealed that new methods were needed to develop high quality training on BPHS for building PHCWs’ capacity; web-based training could be a novel pathway in rural China. The potential barriers and relevant solutions generated by this study should provide guidance to make the training more acceptable to the target study population.

### The importance of BPHS training

Previous studies reported that the PHCWs’ capabilities were insufficient, preventing them from providing adequate services to residents; the quality of public health service is still a major concern in China [6, 8, 40]. Findings from our study complemented the existing literature by providing a similar view that most PHCWs in rural China, especially the village doctors, have insufficient knowledge of BPHS. One cause of the insufficient knowledge is that most of them have a lower educational background [41]. The other cause pertains to the large disparity in salaries, promotion opportunities, and work environments between urban and rural medical institutions. Young medical students or doctors with better educational backgrounds prefer to work in urban medical organizations [7, 42]. Consequently, priorities should be given to building PHCWs’ capacity in rural China to sustainably develop BPHS.

### The deficiencies of face-to-face training on BPHS

The PHCWs’ BPHS training was inadequate and ineffective; this was a significant finding of the study and had not been reported previously. Similar to previous studies, the training method for China’s rural doctors was the face-to-face method concentrated in conference sessions and the training time was inadequate [9–11]. It was difficult to extend the training period in the limited time available and the amount of training content had to be reduced, adversely affecting the training efficacy [43]. Trainees were required to learn content in a limited amount of time, resulting in a discrepancy between their learning needs and preferences and the pace of content presentation. Other limitations related to face-to-face training were also presented, such as inflexibility, time constraints, travel costs, and limited training opportunities [44]. Similar to previous studies, it was difficult for the healthcare workers to make suitable arrangements between work and training [9, 45]. Due to the different knowledge levels and learning needs of the PHCWs, the face-to-face training ignored personal differences and learners’ needs [44]. Challenges in the existing training program suggest that there is an urgent need to develop an innovative and effective training strategy that complements the face-to-face approach to training PHCWs.

### The potential of Web-based training as a method of learning BPHS in rural China

Web-based training might be an effective alternative to overcome the limitations of the existing training strategies in rural settings. Due to the tremendous economic development in China, the number of internet users is approaching 156 million. The internet penetration rate reached 23.7% in rural areas in 2012 [46]. The web has been effectively developed for medical education and health worker capacity training in rural China and other developing countries, such as rural Yunnan province, India, Tanzania, and South Africa [20–23, 27, 28, 30]. Moreover, the majority of PHCWs in the study could easily access a computer and the internet and desired to receive web-based training because of its flexibility, convenience, and cost-effectiveness. Hence, our findings suggest that the internet is a promising tool to deliver knowledge to strengthen the health service capacity of PHCWs in rural China.

### Barriers to the implementation of web-based training on BPHS

Despite the potential of web-based training described herein, several obstacles described in this study should be addressed before applying web technology to BPHS training.

First, low computer literacy of the elderly and inadequate infrastructure had been reported as challenges in resource-constrained countries [20–22]. Age may be an additional factor influencing computer use [47]. Older PHCWs with more years of clinical practice were less likely to access the computer and might not have been familiar with computer and internet use. Recent studies in rural China reported more than 30% of the village doctors in the sample areas (both developed and under-developed regions) were aged 50 or above [48, 49]. As computer literacy is essential to implementing web-based training [21], efforts are needed to help older individuals develop computer skills.

Second, lack of interaction associated with web technology also has been perceived as prevalent and a major drawback [20, 21, 50, 51]. The lack of face-to-face interaction may contribute to professional isolation, a decrease in learning experience quality, unsatisfactory learning outcomes, and a high withdrawal rate from online learning programs [21, 52, 53]. Hence, the PHCWs expressed a strong desire for communication with fellow students and obtaining instruction from experts when studying online.

Third, the societal barriers of lack of time and motivation involve training implementation. Similar to previous studies, time was a common issue for web-based training or education of health professionals [50, 51, 54, 55]. Dickmann *et al*. reported that 29% of the health workers felt that web-based education might increase their workloads [55]. Given their heavy workloads, it might pose a challenge for PHCWs who would need to schedule time to learn how to use a new training website and join a web-based training course while continuing to work. Motivation also was found to be an obstacle in this study and had not been described previously in web-based training for PHCWs in primary care settings. Motivation is an essential element for successful training. It is important to identify potentially compelling reasons for trainees to engage fully in the process [56]. Garrison’s Dimensional Self-Directed Learning Model, which is an integration of self-management, self-monitoring, and motivational dimensions, indicates that self-management and motivation are critical to successful learning [57]. Previous study also revealed that motivation and time management skills are important factors influencing online learning persistence [58].

### Facilitators of implementation of web-based training on BPHS

The facilitators of web-based training, which include computer and internet skills training, user-friendly packages, and technical and administrative support on infrastructure building in the village clinics, *etc*., might mitigate the effects of the above barriers or eliminate them [20, 23, 50, 51]. Several methods (e.g., e-mail, computer conference discussions, telephone, interactive quizzes, forums, pre-post tests, online discussions) reported in previous studies could be used to facilitate trainee-trainee and trainee-trainer communication and prevent or decrease feelings of isolation [21, 51, 52, 54]. Moreover, consistent with previous studies, online training programs should be tailored to the actual needs of the trainees, adapted to fit a country’s healthcare realities, and intended to preserve idiomatic meaning [20, 45, 56]. Finally, incentives such as economic rewards and certificates of completion might promote the enrollment and retention of public health professionals in programs using web-based training strategies [14, 50].

### Strengths and limitations

This study has several strengths. First, it assessed participants’ perceptions about integrating web technology into their BPHS training in rural China’s primary health care settings. Second, the use of FGDs allowed in-depth exploration of the participants' perceptions of web-based training and provided a more detailed report and better understanding of their opinions that would not be gained through quantitative methods alone. The use of qualitative methods prior to the training’s implementation should help identify potential problems and solutions before interventions are needed, which is consistent with recommendations to incorporate qualitative work into intervention development [59, 60].

There are two limitations in this study. First, as this qualitative study and interviews were conducted only in Yichang City, Hubei Province, our findings might not be generalizable to a larger population of PHCWs in rural areas of China. However, data saturation was reached as no new themes or viewpoints were generated. To minimize potential bias in representation, our interviewees included individuals with a diverse set of demographic characteristics and thus varying perspectives about the topic. Second, as is usual in focus groups, we found that some participants were “quieter” on the topic than others were; some of them may also have adjusted their viewpoints to conform to the popular viewpoints. Efforts were made to minimize such bias, including informing participants that there were no “right” or “wrong” answers and encouraging the quieter participants to agree or disagree with any comments. Given the relaxed nature of the FGDs, we believe that this type of bias was minimal in our study. Informed by the findings of this research, follow-up studies might consider applying a quantitative methodology to reach a larger and nationally representative sample.

## Conclusions

As evidenced in the interview data, it is necessary to develop high-quality training programs on BPHS to improve the PHCW’s knowledge and the efficacy of the existing program. The web-based method of training is promising and has the potential to support health workforce capacity building in rural primary healthcare settings. However, focus group participants voiced concerns regarding computer literacy, the effects of the web-based learning method on interaction, time issues, motivation, and inadequate infrastructure. Future studies should focus on when and how to implement web-based training successfully in rural China and how to ensure a beneficial effect on trainees.

## Practice Implications and Future Research

This study reinforced the necessity to embed web-based training into regular training and continuous medical education programs in rural China. To ensure high online training engagement, it is important to identify potential barriers and provide practical solutions prior to implementation. Future research will investigate how to best incorporate these facilitators (e.g., sessions on time management skills, computer usage skill training) into web-based training implementation in primary settings. Effectiveness of the web-based training in rural China should also be explored in the future.

## Supporting Information

S1 Checklist COREQ 32-ITEM Checklist. (DOC)
